# Evidence of SARS-CoV-2 bacteriophage potential in human gut microbiota

**DOI:** 10.12688/f1000research.109236.2

**Published:** 2025-04-23

**Authors:** Mauro Petrillo, Maddalena Querci, Carlo Brogna, Jessica Ponti, Simone Cristoni, Peter V Markov, Andrea Valsesia, Gabriele Leoni, Alessandro Benedetti, Thierry Wiss, Guy Van den Eede

**Affiliations:** 1 Seidor Italy srl, Milano, 21029, Italy; 2 European Commission Joint Research Centre, Ispra, 21027, Italy; 3 Craniomed group srl, Montemiletto, 83038, Italy; 4 ISB Ion Source & Biotechnologies Srl, Bresso, 20091, Italy; 5 International School for Advanced Studies (SISSA), Trieste, 34136, Italy; 6 European Commission Joint Research Centre, Karlsruhe, 76344, Germany; 7 European Commission Joint Research Centre, Geel, 2440, Belgium

**Keywords:** SARS-CoV-2, COVID-19, gut microbiota

## Abstract

**Background:**

In previous studies we have shown that severe acute respiratory syndrome coronavirus 2 (SARS-CoV-2) replicates *in vitro* in bacterial growth medium, that the viral replication follows bacterial growth, and it is influenced by the administration of specific antibiotics. These observations are compatible with a ‘bacteriophage-like’ behaviour of SARS-CoV-2.

**Methods:**

We have further elaborated on these unusual findings and here we present the results of three different supplementary experiments: (1) an electron-microscope analysis of samples of bacteria obtained from a faecal sample of a subject positive to SARS-CoV-2; (2) mass spectrometric analysis of these cultures to assess the eventual de novo synthesis of SARS-CoV-2 spike protein; (3) sequencing of SARS-CoV-2 collected from plaques obtained from two different gut microbial bacteria inoculated with supernatant from faecal microbiota of an individual positive to SARS-CoV-2.

**Results:**

Immuno-labelling with Anti-SARS-CoV-2 nucleocapsid protein antibody confirmed presence of SARS-CoV-2 both outside and inside bacteria. *De novo* synthesis of SARS-CoV-2 spike protein was observed, as evidence that SARS-CoV-2 RNA is translated in the bacterial cultures. In addition, phage-like plaques were spotted on faecal bacteria cultures after inoculation with supernatant from faecal microbiota of an individual positive to SARS-CoV-2. Bioinformatic analyses on the reads obtained by sequencing RNA extracted from the plaques revealed nucleic acid polymorphisms, suggesting different replication environment in the two bacterial cultures.

**Conclusions:**

Based on these results we conclude that, in addition to its well-documented interactions with eukaryotic cells, SARS-CoV-2 may act as a bacteriophage when interacting with at least two bacterial species known to be present in the human microbiota. If the hypothesis proposed, i.e., that under certain conditions SARS-CoV-2 may multiply at the expense of human gut bacteria, is further substantiated, it would drastically change the model of acting and infecting of SARS-CoV-2, and most likely that of other human pathogenic viruses.

## Introduction

In a recent study, we reported a series of observations suggesting unexpected severe acute respiratory syndrome coronavirus 2 (SARS-CoV-2) – bacteria interactions. When cultivated *in vitro* faecal microbiota from infected individuals, we observed sustained growth in SARS-CoV-2 virus copies in 30-day bacterial cultures and SARS-CoV-2-related peptides were detected. ^1^ Furthermore, increase of viral loads was influenced by the administration of specific antibiotics The observations reported in Ref. 1 are in line with additional ones reported more recently, like evidence that gut virome is affected by the SARS-CoV-2 infection and may play an important role in the disease progression of coronavirus disease 2019 (COVID-19), ^2^ the presence of SARS-CoV-2 nucleoprotein (N) in intestinal epithelial cells of approximately 35% of patients with COVID-19 even several weeks or months after initial diagnosis, ^3^ the high rate of positive polymerase chain reaction (PCR) findings in more than one type of clinical specimen collected up to 43 days after COVID-19 patients had presented the first symptom. ^4^

To investigate these observations in more detail, we designed and carried out three different experiments, which make use of electron microscopy (EM), mass spectrometry and next-generation sequencing (NGS). The sample A described in Ref. 1 was used as starting material i.e *.*, an *in vitro* faecal microbiota culture obtained from a stool sample of a SARS-CoV-2 positive individual.

Here we present the results of these experiments, which give evidence of SARS-CoV-2 bacteriophage potential in human gut microbiota.

## Methods

### Ethical considerations

Faecal samples were collected and handled by CranioMed S.R.L. from anonymous donors who agreed to participate in this study by signing informed consent, as foreseen by Italian legislation. No personal information (i.e. age, sex, blood serotype, severity of the disease, time of the collection, fatality, etc.) were collected. The experiments took place in 2021 at the ISB Ion Source & Biotechnologies Srl and Craniomed group srl laboratories, except for Next Generation Sequencing (conducted in service at IGA Tech, Italy). Samples’ preparation for image acquisition and data analyses were performed at JRC in Ispra and in Karlsruhe.

The study is compliant with the JRC Scientific Integrity and Research Ethics guidance.

### Bacterial source and growth

For all three experiments we used, as initial material, the sample A described in Ref. 1 i.e., an *in vitro* faecal microbiota culture obtained from a stool sample of a SARS-CoV-2 positive individual. The bacterial liquid culture derived from sample A, was maintained under stable conditions (here called again sample A). A corresponding negative control was used i.e., an *in vitro* faecal microbiota culture obtained from a faecal sample of a SARS-CoV-2 negative individual (sample B in Ref. 1 and here again called sample B). Bacteria were grown as described in Ref. 1 (see *Materials and Methods*, sections “ *Medium preparation*” and “ *Bacterial culture conditions*”). Briefly, faecal samples (stools) from confirmed COVID-19 affected and healthy individuals were obtained following written informed consent. Stool samples were kept at 4 °C until processing. The NutriSelect ^TM^ Plus nutrient broth (n. 70122-500G, Sigma-Aldrich) was used as bacterial growing broth. The medium was prepared following the protocol recommended by the supplier. Tubes/flasks with growing broth and bacteria were placed in an orbital shaker at 10 g at 37 °C and the liquid culture was left to grow progressively under anaerobic condition.

### Peptide mapping of spike in presence of ^15^N

The sample A bacterial culture was split in two (here called A1 and A2). In total, 0.25 ng of ammonium- ^15^N chloride (>98 % nitrogen-15 isotope ^15^N, >99% (CP), Sigma Aldrich Catalog # 900523) was added to sample A2 as additional source of nitrogen. Measurements of the viral RNA load were performed by using Luminex technology, as described in Ref. 1 (see *Materials and Methods*, sections “ *Determination of viral RNA load*”). Briefly, the detection was performed by using NxTAG ^®^ CoV Extended Panel (Luminex Corporation), a CE-marked (see https://covid-19-diagnostics.jrc.ec.europa.eu/devices/detail/266) real-time reverse transcriptase PCR assay detecting three SARS-CoV-2 genes on the MAGPIX ^®^ NxTAG-enabled System MAGPIX instrument (Luminex Corporation Part Number MAGPIX-XPON4.1-CEIVD), and the AccuPlexTM SARS-CoV-2 Reference Material Kit (SeraCare) as reference standard with sequences from the SARS-CoV-2 genome. Multiplex 96-well plates were *in house* produced and RNA tags were added before the analysis in agreement with manufacturer instructions (Sigma Aldrich, https://www.sigmaaldrich.com/GB/en/product/sigma/luminex) for their use on the MAGPIX ^®^ instrument, and by following the protocol described by Chen *et al.* ^5^ These plates were transferred to a MAGPIX heater at 37 °C, and signal acquisition was performed by using the xPONENT and SYNCT software (Luminex Molecular Diagnostics) using a commercially available reference standard with sequences from the SARS-CoV-2 genome (AccuPlex ^TM^ SARS-CoV-2 Reference Material Kit, SeraCare).

At day +7 from ^15^N addition, aliquots from A1 and A2 were analysed to detect SARS-CoV-2 spike protein by peptide mapping, performed by using Surface-Activated Chemical Ionization/Electrospray Ionization mass spectrometry (SACI/ESI-MS) technology. The approach uses an ORBITRAP MS (ThermoFisher, San Jose, USA) coupled to a surface activated chemical ionization (SACI) /ESI source, and operated in positive ion mode. SARS-CoV-2 Spike Protein S1/S2 (ThermoFisher, Catalog # RP-87680) was used as an analytical standard. The characterisation of viral peptides was conducted as described in Ref. 1 (see *Materials and Methods*, section “ *Characterisation of viral peptides by Mass Spectrometry (MS)*”). The following MS parameters were used: direct infusion flow rate, provided by means of a syringe pump, was set to 5 μL/min, with additional solvent provided at a flow rate of 100 μL/min. by an in-line HPLC pump; the N _2_ drying gas temperature was 350°C; the N _2_ nebulizing gas flow rate was 12 L/min. Two microscans (average) were acquired.

The ionization source parameters were: ESI ionization voltage = 3,000 V, applied via a home-built external power supply to the spray needle; APCI needle current = 3,000 nA; SACI surface potential = 47 V for the in-source ionization, with an inter-capillary voltage gradient between –100 and –1,500 V, being employed to produce the CIMS effect; ion-source pressure = 2 bar. Pressure was varied by providing N2 gas via a tube connected to the ion source, and it was monitored by using a manometer (Stanley, Torino, Italy).

### Samples preparation for electron microscopy image acquisition and analysis

Aliquots of sample A and corresponding negative control were collected and fixed overnight at 4 °C in Karnovsky 2% fixative freshly prepared in the laboratory using paraformaldehyde (Sigma Aldrich Catalog # 16005) 2% (w/v) in Na-Cacodilate (Sigma Aldrich Catalog # 233854) 0.05 M pH 7.4 and glutaraldehyde (Sigma Aldrich Catalog # G5882) 2% (v/v). Once fixed, they were washed 3 times with 0.05M Na-Cacodilate solution at pH 7.3, dehydrated in a graded series of ethanol (Sigma Aldrich Catalog # 493546) solutions in MilliQ water (30%-50%-75%-95% (v/v)) for 15 min. each, and 100% for 30 min.; 3 μL of suspension were deposited on Formvar Carbon coated 200 mesh copper grids (Agar Scientific, USA) and dried overnight in a desiccator. Some grids were directly analysed by JEOL JEM-2100 High Resolution-transmission electron microscope (TEM, JEOL, Italy) coupled with energy dispersive X-ray spectroscopy (EDX Bruker, Italy) at 120 kV, in TEM mode at magnification from ×3,000 to ×80,000 for imaging, and at 120kV magnification of ×80,000 and ×300,000 in STEM-Hypermap mode acquisition for elemental analysis. Some grids were immuno-labelled by rabbit polyclonal to SARS-CoV-2 nucleocapsid protein antibody (Abcam Catalog # ab273167) and anti-rabbit polyclonal IgG (whole molecule)-Gold 10 nm secondary antibody produced in goat (Sigma Aldrich catalog # G7402) followed by 5 min. uranyl acetate (Fluka catalog # Fluka 7394) 5% (w/v) stain. Sample A and the corresponding negative control were also immuno-labelled and embedded in epoxy resin (Sigma Aldrich, Epoxy Embedding Medium kit catalog # 45359) to prepare ultrafine slices for analysis as follows: both were fixed with a solution of 4% (v/v) paraformaldehyde and 0.1% (v/v) glutaraldehyde in 0.2 M 4-(2-hydroxyethyl)-1-piperazineethanesulfonic acid (HEPES, ThermoFisher Scientific catalog # J61017.AK) buffer for 15 min. and washed HEPES buffer two times. Samples were incubated in 4% (v/v) paraformaldehyde for 30 min. and washed 3 times for 5 min. each with phosphate buffered saline without CaCl _2_ and MgCl _2_ solution (PBS, ThermoFisher Scientific, Gibco catalog # 70011-36). Samples were incubated with block-solution (50 mM NH _4_Cl, 0.1% v/v saponin, 1% v/v Bovine Serum Albumin (BSA) in PBS, Sigma Aldrich catalog # 09718; # 47036; # B6917, respectively) for 30 min., and then with rabbit polyclonal to SARS-CoV-2 nucleocapsid protein primary antibody (Abcam Catalog # ab273167) diluted in block solution 1:100 (overnight). Pellets were washed 6 times for 2 min. each with PBS. Diluted Nanogold ^®^-Fab’ (goat polyclonal) or anti-rabbit IgG (Nanoprobes, catalog #2004) Fab fragments 1:100 in block solution was added to pellets, which were then incubated for 2 hours. Pellets were washed 6 times for 2 min. each with PBS and 3 times for 5 min. each in distilled water. Pellets were incubated with gold-enhancement mixture Gold-enhance EM kit (Nanoprobes catalog # 2113) for 5 min. and then washed 5 times for 5 min. each with distilled water and 3 times for 5 min. each with PBS.

Pellets were post-fixed with the mixture of 2% (v/v) OsO _4_ (Sigma Aldrich catalog # 75632) and 0.1M PBS (pH 6.8) for 25–30 min. on ice and washed 3 times with water.

Thiocarbohydrizide 1% (w/v) in water (Sigma Aldrich catalog # 223220) was added for 5 min., followed by washing (3 times with water).

After, pellets were treated with a mixture of 2% (v/v) OsO _4_ and 3% (w/v) potassium ferrocyanide (Sigma Aldrich catalog # P9387) (1:1) for 25 min., and washed 3 times with water. Uranyl acetate (0.5% w/v) diluted in water was added, and pellets were stored overnight at +4 °C, then washed with water 6 times. Dehydration was conducted in a graded series of ethanol solutions from 50%, 70%, 90% (v/v) ethanol every 10 min. to 100% ethanol for 30 min., changing the 100% ethanol solution every 10 min. Mixture of epoxy resin was added to samples for 4 hours at room temperature. Specimens were incubated at 60 °C in the oven for 24 hours to allow resin polymerization. Ultrathin sections (50-70 nm) were obtained using Leica EM UC7 ultramicrotome (Leica, Italy) and stained for 25 min. with uranyl acetate (Fluka catalog # Fluka 7394) 5% (w/v) and freshly prepared Reynold’s lead citrate solution for 20 min., washed and dried. They were then collected on Formvar Carbon coated 200 mesh copper grids (Agar Scientific, USA) and imaged by TEM, as described above. Sample A and corresponding negative control were analysed by scanning electron microscopy (SEM) (Nova600i Nanolab, ThermoFisher, Eindhoven) using an acceleration voltage of 2 and 5 kV and the in-lens detector. The particles present in each of the analysed sample (independently of their nature) are irreversibly immobilized on a chip by self- assembling, due to electrostatic forces. The particles are separated and distributed randomly on the chip surface, facilitating the SEM characterization. The chip consists of a 0.025 mm ^2^ silicon chip coated with a 70 nm layer of polytetrafluoethylene (PTFE) and subsequently with a monolayer of poly (diallyldimethylammonium chloride) (PDDA) which confers a positive charge to the surface. Details on the preparation of the chip can be found in Ref. 6. The chip is immersed overnight, and it can immobilize all the negatively charged particles present in the liquid sample. Then it is removed, thoroughly rinsed in ultrapure water, and dried under a gentle N _2_ flow. A very thin (<10 nm) layer of carbon is deposited on the chip prior the SEM analysis.

### Plaque assay

*Faecalibacterium prausnitzii* and *Dorea formicigenerans* strains were purchased at the Leibniz Institute DSMZ-German Collection of Microorganisms and Cell Cultures (DSM Numbers 17677 and 3992, respectively). They were grown in liquid broth according to the information provided by the Leibniz Institute DSMZ (available at https://www.dsmz.de/collection/catalogue/details/culture/DSM-17677 and https://www.dsmz.de/collection/catalogue/details/culture/DSM-3992, respectively), and after 7 days transferred on agar plates and grown at the same conditions (37 °C, humidity 95%, CO _2_ 5%). According to the information provided by the Leibniz Institute DSMZ, they have been both isolated from human faeces.

After 3 days, plates were fully covered by bacteria. Each plate was schematically split in four areas and inoculated with: 0.40 μL double distilled water; 0.40 μL of a control supernatant negative to SARS-CoV-2; two aliquots (0.40 μL each) from two different supernatants positive to SARS-CoV-2. Aliquots of newly formed plaques were collected to perform NGS.

### Next Generation Sequencing

Collected samples were centrifuged for 3 minutes at 3,000 rcf at room temperature. Nucleic acids were extracted from the supernatant by using the PureLink™ Viral RNA/DNA Mini Kit (ThermoFisher, Waltham, MA, United States) according to the manufacturer’s instructions. An amount of 10 μL of obtained total RNA was retrotranscribed using random hexamers, RNaseOUT™ Recombinant Ribonuclease Inhibitor and SuperScript™ II Reverse Transcriptase (all Life Technologies - Invitrogen), according to the manufacturers’ instructions. The SARS-CoV-2 genome was sequenced in service at IGA Tech (Italy) using a proprietary two-step PCR protocol for the amplification: 1) tiling in overlapping fragments of ~250 bp, 2) amplicon indexing. Libraries were sequenced on an Illumina NovSeq6000 platform, PE 150 bp mode.

### Raw data quality control

FastQC (version 0.11.9, default parameters) (RRID:SCR_014583) ^7^ was used to obtain quality metrics from all sequencing raw data. Through FastQ Screen (version 0.14.1, default parameters) (RRID:SCR_000141) ^7^ and the MultiQC (version 1.11, default parameters) (RRID:SCR_014982) ^8^ tool run on the Galaxy server (RRID:SCR_006281), ^9^ outputs were inspected, and no sequencing issues were found from these quality checks. A summary of the quality checks and sequencing is provided in Figure S3.

### Mapping and variant calling

Mapping of reads and variant calling were conducted on the Galaxy server by running the workflow “SARS-CoV-2 Illumina Amplicon pipeline - iVar based”. ^10^ Obtained consensus sequences were compared to reference by running COVID-Align. ^11^ Amino acid changes variations were identified at protein level by running Nextclade. ^12^ The comparison with known variations was made with respect to GISAID data (RRID:SCR_018279), ^13^ analysed by CoV-GLUE-Viz (update 15/09/2021). ^14^

## Results

To investigate more in detail our previous observations reported in Ref. 1 indicating SARS-CoV-2 – bacteria interaction, we designed and carried out three different experiments:

1. *In vitro* faecal microbiota from a SARS-CoV-2 positive individual (here called sample A) was grown in liquid culture and collected aliquots were analysed by EM image analysis to visualize the eventual presence of SARS-CoV-2 and to get some insight in the eventual interaction with bacteria.
2. Natural nitrogen-14 ( ^14^N) and nitrogen-15 ( ^15^N) isotopes, usually employed in metabolomics, proteomics and genomics functional studies, ^15^^,^^16^ were used to monitor the virus proliferation. Sample A was grown in presence of ^14^N, and then ^15^N was added. Presence of ^15^N included in the spike protein was tested by performing peptide mapping analysis.
3. *Faecalibacterium prausnitzii,* member of *Oscillospiraceae* family (class *Clostridia*, NCBI:txid 853) and *Dorea formicigenerans*, member of *Lachnospiraceae* family (class *Clostridia*, NCBI:txid 39486) had previously been reported as being strongly reduced in COVID-19 patients. ^17^^,^^18^ For this reason, these two bacterial species were used for plaque assays to test aliquots of supernatants from sample A. Observed plaques were collected and further processed to perform NGS.

We conducted all three experiments using, as initial material, the sample A described in Ref. 1 i.e *.*, an *in vitro* faecal microbiota culture obtained from a stool sample of a SARS-CoV-2 positive individual. The bacterial liquid culture derived from sample A (here also called sample A), was maintained under stable conditions. A corresponding negative control was used i.e., an *in vitro* faecal microbiota culture obtained from a faecal sample of a SARS-CoV-2 negative individual, called sample B (see Ref. 1 for details).

### Electron microscopy image analyses reveals the presence of virus-like particles inside and outside bacteria

Aliquots of sample A and sample B (as negative control) were collected and analysed by SEM and by transmission electron microscope (TEM) coupled with energy dispersive X-ray spectroscopy.

**Figure 1.**
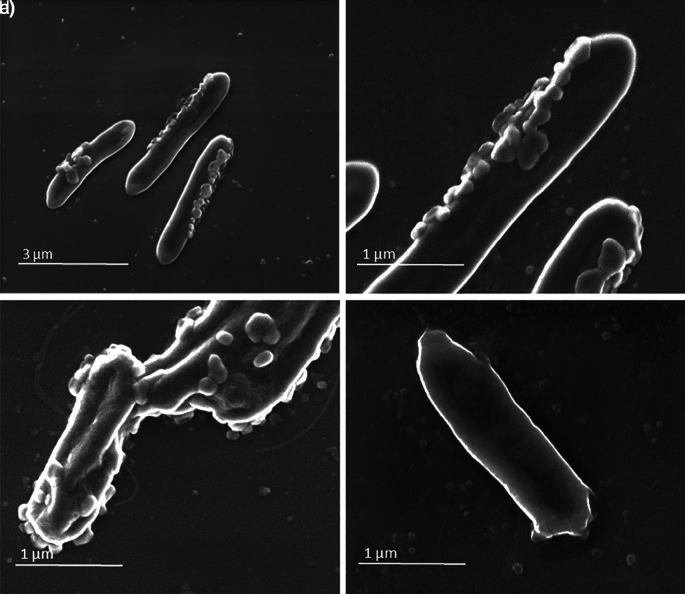
Sample A and sample B images obtained by scanning electron microscope (SEM). Panels a (magnification ×20,000), b (magnification ×80,000), and c (magnification ×80,000) (sample A) show virus-like particles on bacteria surface from sample A (i.e., *in vitro* faecal microbiota culture obtained from a SARS-CoV-2 positive individual). Panel d (magnification ×80,000) shows the sample B (i.e., *in vitro* faecal microbiota culture obtained from a faecal sample of a SARS-CoV-2 negative individual) used as control.

**Figure 2.**
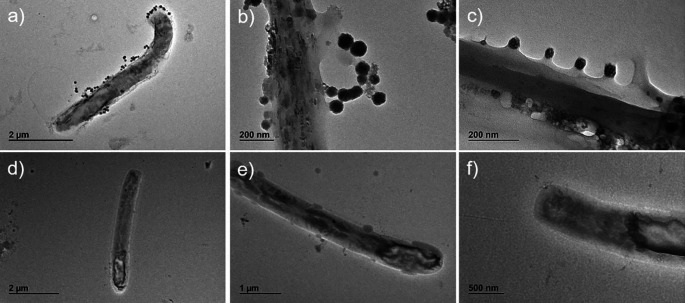
Sample A and sample B images obtained by transmission electron microscope (TEM). Panels a (magnification ×4,000), b (magnification ×20,000), and c (magnification ×30,000) (sample A, described in Figure 1) present whole bacteria fixed and dehydrated and show the presence of virus-like particles on bacteria surface strictly attached and interacting with the wall. Panels d (magnification ×3,000), e (magnification ×5,000), and f (magnification ×8,000) present whole bacteria not exposed to virus-like particles in sample B (described in Figure 1) used as control.

SEM analysis showed in sample A the presence of virus-like particles on the bacteria surface ( Figure 1, panels a, b, c). This observation was also confirmed by TEM analysis ( Figure 2, panels a, b, c) in which round virus-like particles of 25–100 nm size are clearly visible. In the SEM images, on carbon-coated samples, we observed many particles with round morphology attached to the bacteria wall. These round structures were not visible on bacteria from the control samples ( Figure 1, panel d), which instead showed regular smooth surface. In the TEM analysis, particles darker than the substrate, showing rough surface, regular and spherical morphology are clearly visible and distinguishable from the bacteria structure. The particles are clearly interacting with the bacterial wall forming a sort of a bridge ( Figure 2, panel b) and attached in different places of the wall ( Figure 2 a, b, c).

**Figure 3.**
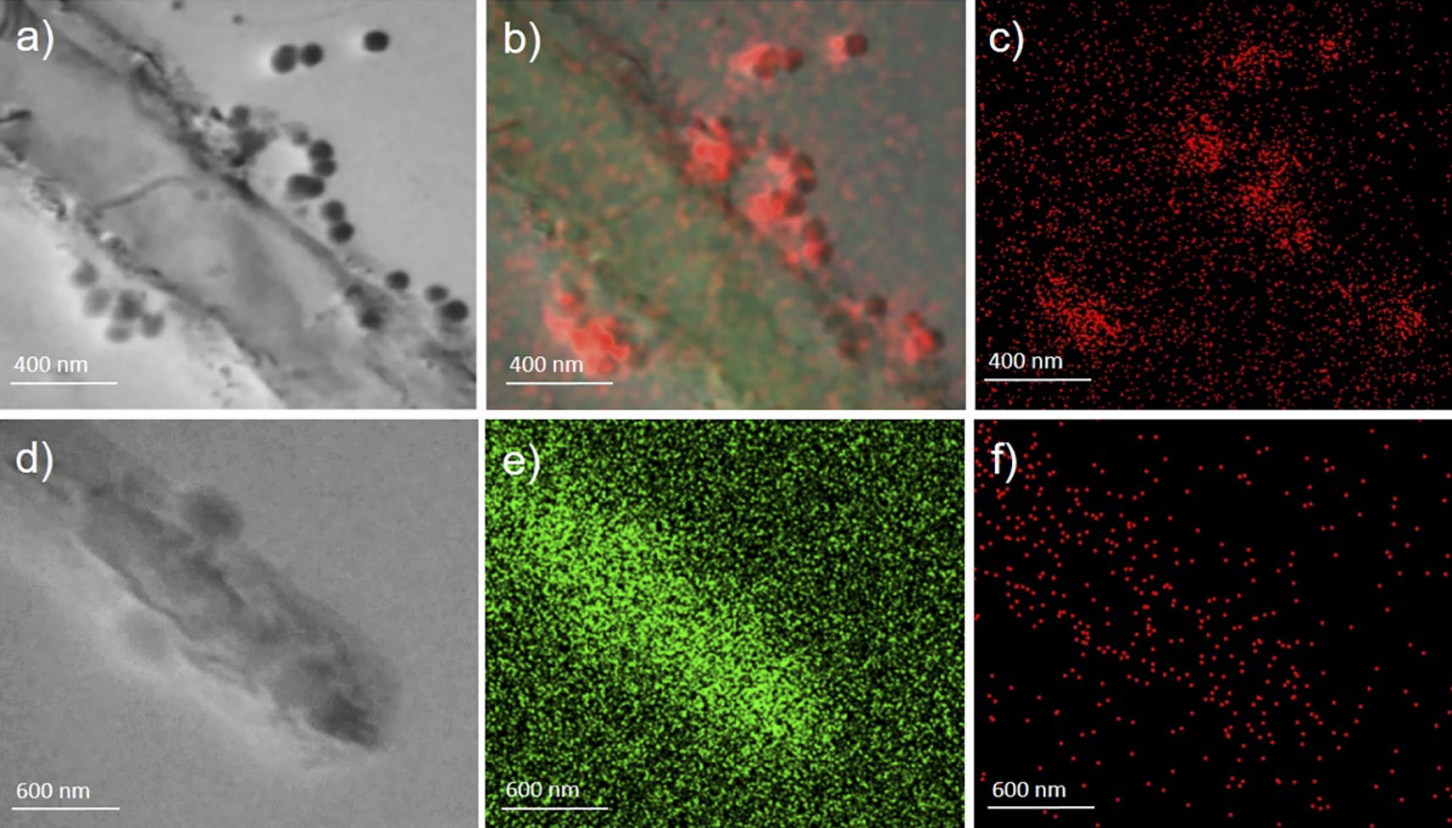
Sample A and sample B images obtained by transmission electron microscope coupled with energy dispersive X-ray spectroscopy (STEM-EDX). Panels a, b, and c (magnification ×80,000) (sample A, described in Figure 1) present whole bacteria fixed and dehydrated in the presence of virus-like particles on their surface strictly attached and interacting with the wall (panel a, acquisition in bright field). Each particle contains a high amount of phosphorus (red in panels b) as compared to the background; carbon is also present in (green in panel b) Panels d, e, and f (magnification ×60,000) show the same acquisition made on sample B (described in Figure 1) used as control (d=bright field; e=carbo; f=phosphorus).

In a subsequent step, employing immuno-labelling of the grid, the presence of SARS-CoV-2 nucleocapsid protein in sample A was confirmed ( Figure 3, panel a). In addition, high amount of phosphorus (P) ( Figure 3, panels b-c) is measured, as well as calcium and zinc (Ca and Zn, see supplementary Figure S1 in the *Underlying data* ^38^) in comparison to the background, which suggests that the observed structures are rich of P, which is negatively charged and thus it might absorb from the surrounding micro-environment positive charged ions, which is also in line with the observed lower levels of Ca and Zn in COVID-19 patients. ^19^^–^^21^

**Figure 4.**
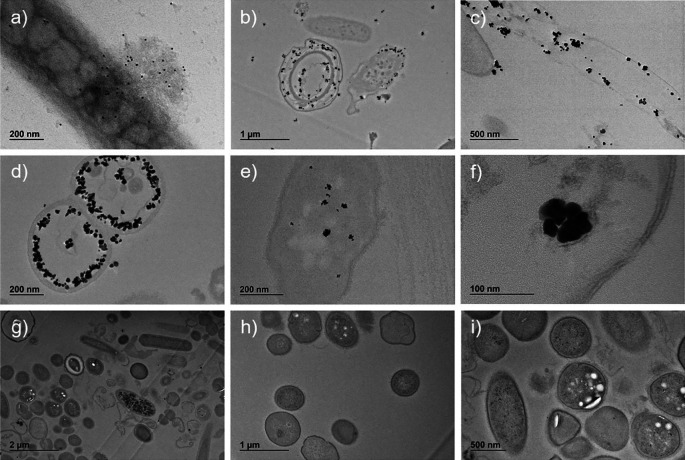
Sample A and sample B images obtained by transmission electron microscope (TEM) with immuno-labelling of SARS-CoV-2 nucleocapsid protein. Presence of SARS-CoV-2 nucleocapsid protein conjugated with secondary Ab-gold 10 nm in whole bacterium (panel a, magnification ×20,000) and conjugated with secondary Ab NANOGOLD enhance in bacteria ultrafine slices (panels b, magnification ×6,000; c, magnification ×12,000; d, magnification ×20,000; e, magnification ×25,000; f, magnification ×80,000) from sample A (described in Figure 1). Ultrafine slices of negative control (Sample B, described in Figure 1) do not show significant presence of SARS-CoV-2 nucleocapsid protein (panels g, magnification ×3,000; h, magnification ×6,000; i, magnification ×8,000).

Immuno-labelled ultrafine slices of bacteria in sample A confirmed the presence of SARS-CoV-2 nucleocapsid protein both outside and inside bacteria ( Figure 4, panels b-f ).

The qualitative analysis of ultrafine slices showed the presence of SARS-CoV-2 nucleocapsid protein in bacteria with different grades of the ultrastructure damage, as compared to the negative control. Most of the protein was free in the cytoplasm and in some cases surrounded by a membrane ( Figure 4, panel f ). The size and morphology of the enhanced gold signal particles were similar to what was observed in the whole bacteria samples.

The same analyses performed on the negative controls ( Figure 1, panel d, Figure 2, panels d-e-f, Figure 3, panels d-e-f, and Figure 4, panels g-h-i) did not reveal any comparable signal.

These images show the presence of virus-like particles inside and outside bacteria.

### Bacterial growth in presence of ^15^N detected *de novo* synthesis of spike protein

The experiment comprised of: (1) splitting of Sample A into two aliquots (here called A1 and A2), followed by addition of ammonium- ^15^N chloride to sample A2 as a supplementary source of nitrogen (day 0); (2) growth of A1 and A2 for 7 days; (3) measurements of the viral RNA load by Luminex technology at day 0 and day 7. (4) peptide mapping analysis by mass spectrometry at day 7.

**Figure 5.**
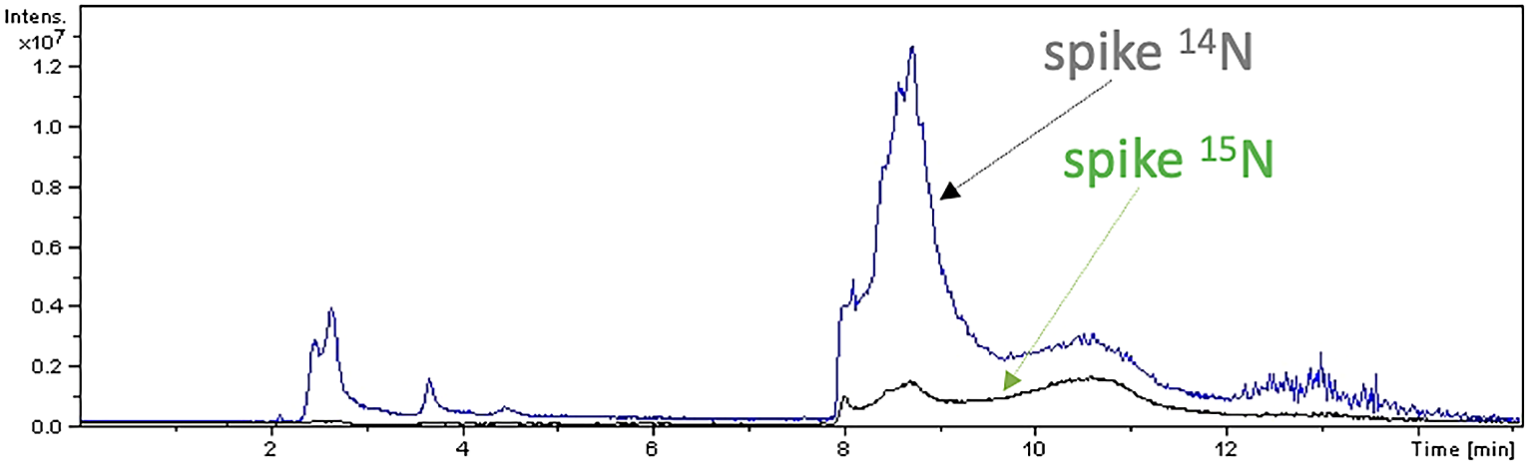
Peptide mapping of SARS-CoV-2 spike protein. Peptide mapping of SARS-CoV-2 spike protein were acquired by means of liquid chromatography mass spectrometry associated to ^14^N and ^15^N profiles, and performed on an aliquot of sample A2.

**Figure 6.**
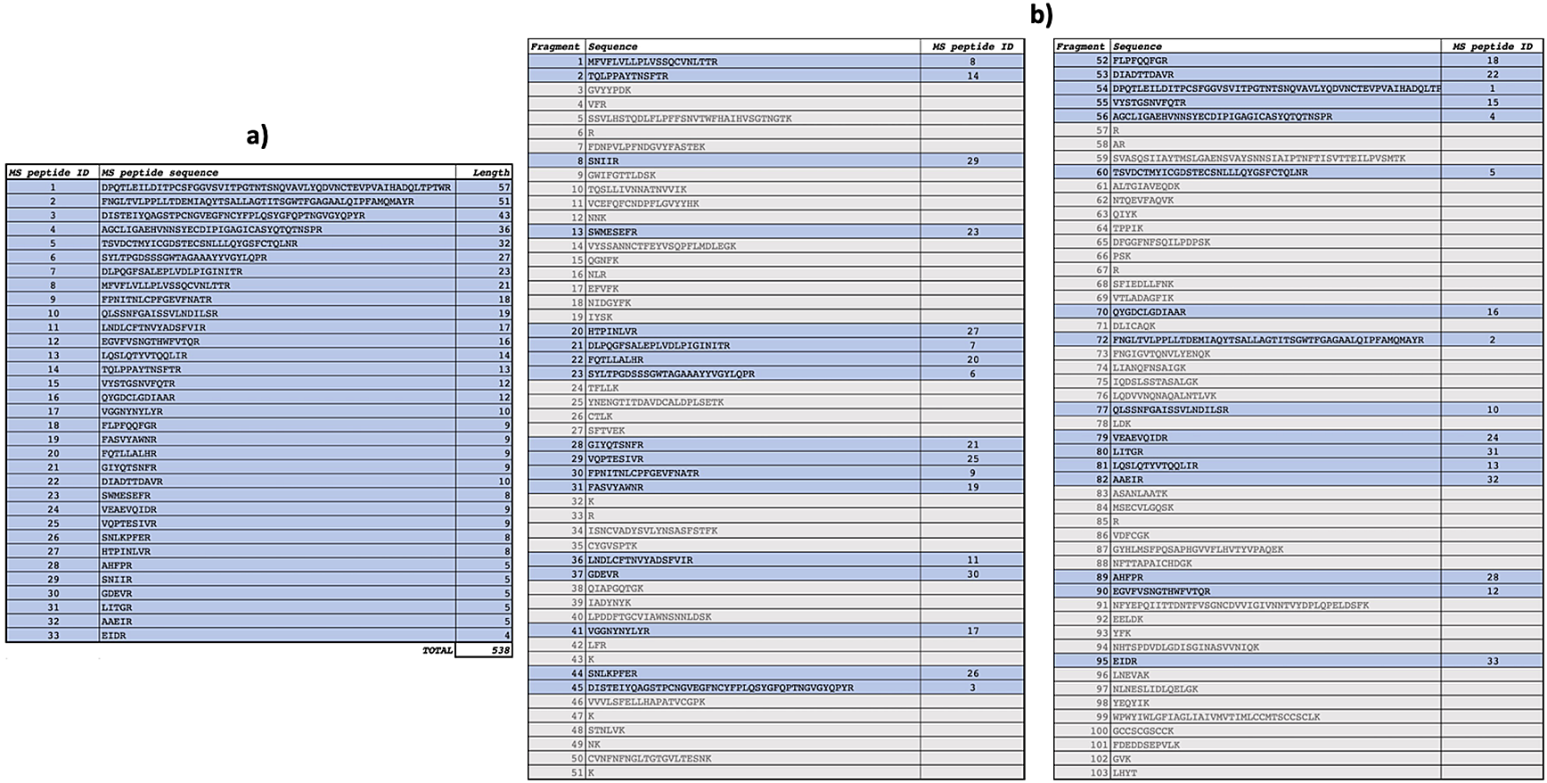
Characterization of spike peptides by Mass Spectrometry. Identified spike peptides labelled with ^15^N and mapping on the reference spike protein (NCBI Reference Sequence: YP_009724390.1) are listed (panel a). They correspond to fragments obtained from trypsin digestion of the reference spike protein (panel b).

Peptide mapping analysis revealed incorporation of ^15^N in spike protein: after 7 days, spike labelled with ^15^N ( Figure 5) and spike labelled with ^14^N- ^15^N were detected in sample A2, but not in sample A1. Sequences of ^15^N-labelled spike peptides were determined ( Figure 6, panel a) and mapped ( Figure 6, panel b) on the SARS-CoV-2 reference spike protein (NCBI Reference Sequence: YP_009724390).

Increase of viral RNA load was also observed in both A1 and A2, as already observed previously in Ref. 1.

These results indicate *de novo* synthesis of SARS-CoV-2 spike protein in human gut bacteria.

### Plaque assays to confirm sensitivity of commensal gut bacteria to SARS-CoV-2

*F. prausnitzii* and *D. formicigenerans* strains were individually grown in liquid broths. After 7 days, bacteria from each broth were transferred on agar plates and grown under the same conditions. When plates obtained full bacterial growth cover (i.e *.*, 3 days later), plates were each schematically split in four sectors, and inoculated with:

• Aliquots of two different supernatants positive to SARS-CoV-2 – one per sector.
• An aliquot of a control supernatant negative to SARS-CoV-2.
• An aliquot of double distilled water.

**Figure 7.**
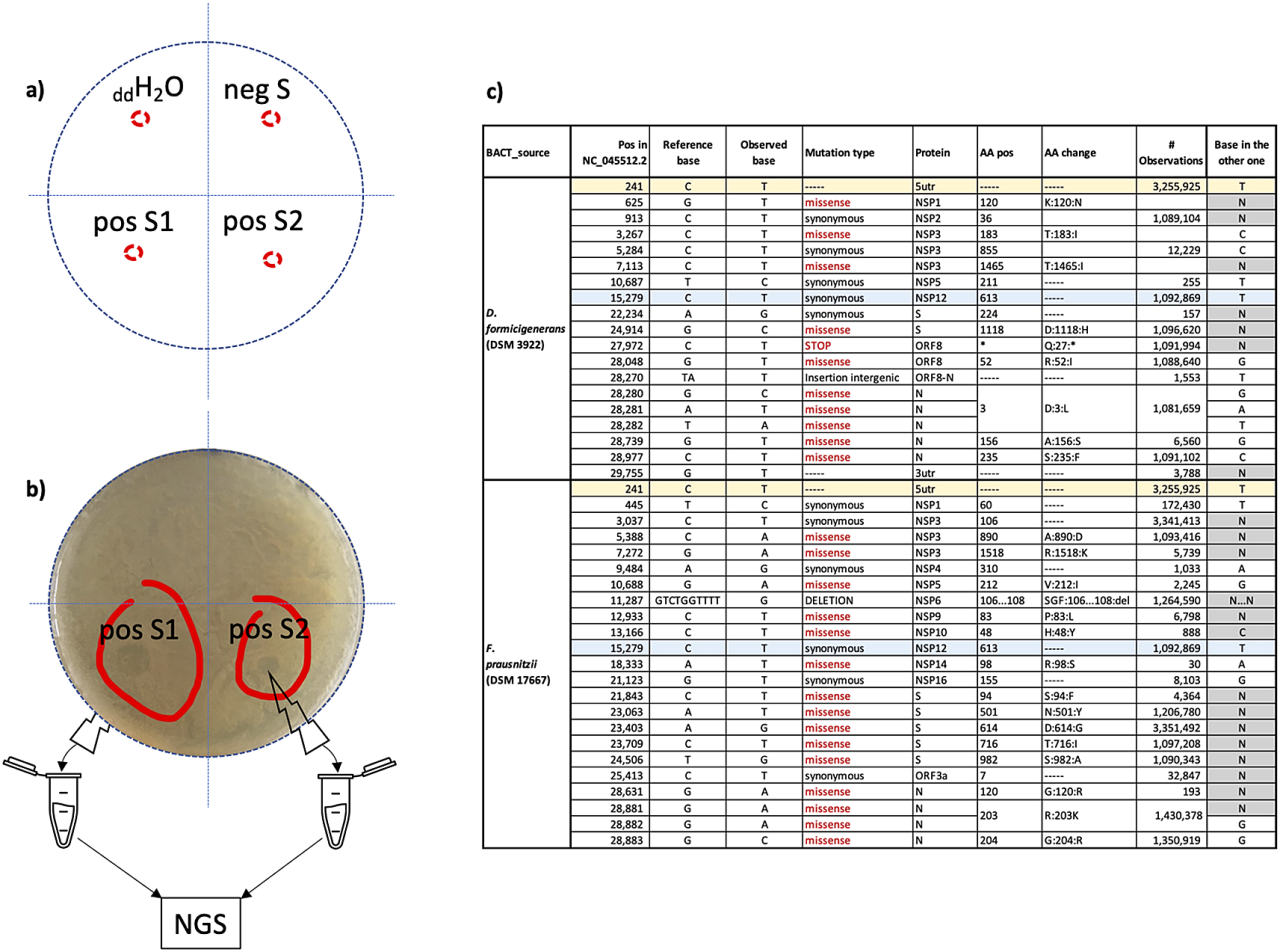
Plaque assay. a) schematic split of the plates used in the assay in four sectors: _dd_H _2_O=double distilled water; negS = control supernatant negative to SARS-CoV-2; pos S1 and pos S2 = supernatants positive to SARS-CoV-2. b) observed plaques (red circled) were individually collected to perform NGS experiments. c) sample-specific nucleic acid changes identified in the two distinct sequence sets (column “BACT_source”): column “# Observations” represents the number of sequences in GISAID that, at the time of the analysis (15/9/2021) showed the same base change; column “base in the other one” indicates the base identified at the same position in the other sequence set; changes found in both sets are highlighted as rows with the same colour.

No plaque was observed in the areas where aliquots of double distilled water and of the control supernatant negative to SARS-CoV-2 where added ( Figure 7, panels a), sectors *_dd_H _2_O and negS* and corresponding in panel b)). On the contrary, plaques were observed in the areas where aliquots from two different supernatants positive to SARS-CoV-2 were added ( Figure 7, panels a) and b), sectors *pos S1* and *pos S2*).

Aliquots of plaques spotted on *F. prausnitzii* and *D. formicigenerans* growth were collected to perform NGS experiments. Obtained reads from *F. prausnitzii* and *D. formicigenerans* phage plaques map and cover 62% and 48% of the SARS-CoV-2 genomic reference sequence (NCBI Reference Sequence: NC_045512), respectively. Overviews on the quality of the sequencing experiments are provided in the *Underlying data* (as Figure S2), together with the obtained raw reads, two distinct SARS-CoV-2 consensus genomic sequences, and with the alignment of these sequences to the SARS-CoV-2 genomic reference sequence. ^38^

Many regions of SARS-CoV-2 were sequenced in both samples, and bioinformatics analyses (variant calling) revealed the presence of sample-specific nucleic acid changes i.e *.*, present in reads sequences from only one of the two grown bacterial species. Identified variations are reported in Table 1: only two of them are common. All nucleic acid changes except one (see Figure 7, panel c) were already observed in other SARS-CoV-2 genomic sequences deposited in GISAID.

These results give evidence of SARS-CoV-2 bacteriophage potential in the two bacterial species tested and normally present in human gut microbiota.

## Discussion

We present a series of experiments to better understand previous observations that suggested a bacteriophage-like behaviour of SARS-CoV-2 in growing faecal microbiota derived from SARS-CoV-2 infected individuals. ^1^

Firstly, by electron microscopy image analyses, we observed virus like structure of around 25–100 nm size interacting with the outside of the wall of bacterial cells and found inside bacteria. The immuno-labelling with Anti-SARS-CoV-2 nucleocapsid protein antibody confirms the presence of SARS-CoV-2 both outside and inside bacteria. In addition, high levels of P, Ca and Zn were measured where spherical structures were observed. This is in line with previous findings that report spike and nucleocapsid proteins as highly phosphorylated ^22^ and patient affected by severe COVID-19 depleted in Zn ^19^ and Ca. ^20^

Secondly, drawing inspiration from the Meselson and Stahl experiment, who used two isotopes of nitrogen i.e., the common light ^14^N, and the rare and heavier ^15^N, as sources of nitrogen to demonstrate semi-conservative DNA replication, ^23^ we used the same in *in vitro* faecal microbiota cultures obtained from a stool sample of a SARS-CoV-2 positive individual to assess *de novo* synthesis of SARS-CoV-2 spike protein.

^15^N-labelled spike protein was detected only in presence of ^15^N as additional source of nitrogen and not in control sample (where only ^14^N-labelled spike was identified). Such a synthesis went in parallel to an increase of the viral RNA load, in line with what already observed previously in. ^1^ These results provide evidence that SARS-CoV-2 RNA is both replicated and translated in the bacterial cultures of faecal origin.

Finally, by performing plaque assays with aliquots of supernatants from cultures of faecal microbiota derived from SARS-CoV-2 infected individuals on pure cultures of two faecal anaerobic bacteria ( *F. prausnitzii* and *D. formicigenerans*) reported to be strongly reduced in COVID-19 patients, we observed the formation of plaques characteristic for the presence of phages.

RNA was extracted and NGS sequenced from aliquots individually collected from the plaques. The reads obtained from *F. prausnitzii* and *D. formicigenerans* viral plaques mapped a span of 62% and 48% of the SARS-CoV-2 genomic reference sequence respectively (NCBI Reference Sequence: NC_045512). Bioinformatic analyses revealed the presence of nucleic acid differences in comparison to the SARS-CoV-2 reference sequence. Interestingly, the two sets of bacterium-specific reads each displays a specific nucleic acid polymorphism signature, potentially suggesting different replication environment in the two bacterial cultures.

The SARS-CoV-2 genome was not entirely sequenced, probably due to the chosen sequencing protocol, which (like any other protocol of sequencing SARS-CoV-2 genome) is not optimised for bacterial phage plaques from agar plates as starting material. Further sequencing experiments will be performed to demonstrate reproducibility. However, the two independent experiments we performed (each with a confirmed negative control), gave similar results. Each of them yielded NGS-derived SARS-CoV-2 genome sequences, with distinct nucleic acid variations, all of which have already been reported in other SARS-CoV-2 sequenced genomes, as reported in Figure 7, panel c.

The presence of identified variations not specific of any lineage, and the existence of culture-specific polymorphisms are in line with the intra-host *quasispecies* diversity of SARS-CoV-2, recently reported at both nucleic acid ^24^ and amino acid level. ^25^ In our experiments, these observations might be either a consequence of different lineages already present in the supernatant aliquots of infected bacteria used in the plaque assay, or a result of the two different bacterial “cellular machinery” environments, which the virus used when infecting two different species. Both consequences cannot be excluded. However, the presence of many identified variations not specific of any lineage, provides an indication that bacteria might be a potential source of novel SARS-CoV-2 mutations, and gives rise to the possibility that intra-host SARS-CoV-2 haplotypes might reflect different intestinal bacterial prior host environments of the virus. This observation would represent the basis for one of the proposed origins of the Omicron variant ^26^ i.e *.*, that Omicron (and other variants) might have evolved in the gut bacteria of one person. Linear regression models, like those used to dissect evolution, mode of transmission, and mutational landscape of SARS-CoV-2 variants ^27^ should consider the role of multiple bacterial hosts living in one infected holobiont. Likewise, investigations about the origin of the COVID-19 pandemic should add new key questions ^28^ in light of this. Since their discovery, interactions of phages with bacteria have been studied mainly in pairwise experiments. Only in recent years, growing numbers of studies have focused on the coevolution of phages and bacteria in communities, revealing the complexity of phage–bacterium interactions and evolution of phages in bacteria. ^29^ Wylezich *et al.* failed to identify tissue specific genetic adaptation of the virus in COVID-19 patients’ upper and lower respiratory tract. ^30^ One explanation might be that all cells of the same individual differ much less in terms of their replication or translation machinery, than taxonomically remote bacterial cells like those present in the human gut microbiota. In light of this, intra-bacterial diversity generation of the virus might potentially be more relevant than that occurring during human cells infection.

Our results strongly suggest interactions of SARS-CoV-2 with bacteria in intestinal microbiota. The findings that SARS-CoV-2 is able to enter the cells of bacteria, which are part of the normal human gut microflora and is able to synthesize both the nucleic acid and viral peptides brings about a wide range of important potential implications for human health and particularly for the management and the control of the spread of this virus. For example, the ability of SARS-CoV-2 to infect and replicate in human gut bacteria could induce a situation where infected bacteria can harbour the virus for extended periods of time, potentially long after systemic infection in the human host has already been cleared. From an epidemiological point of view, this could contribute to much longer, potentially very long-term period of viral shedding through the faecal route, with a possible increased risk of onward transmission. Furthermore, prolonged persistence of a replicating virus in the intestinal lumen would render periodic renewals of systemic SARS-CoV-2 infection in the individual possible, thus potentially causing a relapse of COVID-19 without external reintroduction. All of these possibilities taken together would contribute to the augmentation of R _0_ – the basic reproduction number of the virus, primarily through extending the duration of infectiousness component of R _0._^31^

Gastrointestinal involvement is a frequent feature of long COVID-19 syndrome, ^32^ furthermore long-term presence of SARS-CoV-2 nucleocapsid protein was found in the gut mucosa of patients suffering from long COVID-19. ^33^ Our finding that intestinal bacteria may support SARS-CoV-2 replication therefore is likely to have important bearing on explaining the pathophysiology of the gastrointestinal symptoms during long COVID-19. On a separate note, the evidence we provide here that SARS-CoV-2 is capable of replicating in *F.prausnitzii* gives new significance to earlier evidence of inverse correlation between abundance of this anti-inflammatory bacterium in the intestines and COVID-19 severity, ^17^ suggesting indirectly that infection of symbiotic gut bacteria may correlate positively with disease severity. It is important to highlight that this study is limited to SARS-CoV-2 interaction with only two bacterial strains, i.e., *F. prausnitzii* and *D. formicigenerans*, chosen because both previously reported as strongly reduced in COVID-19 patients. ^17^^,^^18^ These are common gram-positive anaerobic commensals in the human gut, and thus it is likely they were also present in the faecal samples collected and used in this study. The interaction of SARS-CoV-2 with other common commensals, such as the facultative anaerobe gram-negative *Escherichia coli* should be investigated, too.

At the population level, the ability of the virus to persist in gut bacteria may shed new light on the otherwise puzzling long-term stability of viral RNA in wastewater and public sewers, a phenomenon successfully used for local early warning SARS-CoV-2 surveillance. ^34^ Another aspect, related to the above is the possibility of replication of SARS-CoV-2 in bacteria outside of the human body – in the environment or in the gut or other microflora of other vertebrate hosts. From the point of view of epidemiological control, one of the fundamental obstacles to SARS-CoV-2 elimination is its ability to infect and be maintained as an epidemiological reservoir in multiple vertebrate species. ^35^ Thus, even if the elimination effort in humans was successful, frequent reintroductions of the infection from reservoir species could reverse the success. In this context, maintenance of the virus in bacteria in the environment could add one further potentially large and ubiquitous epidemiological reservoir for potential reintroduction of the infection back to humans or animal reservoirs. In the same vein, if SARS-CoV-2 can persist or replicate in bacteria in the human microbiota, such could also be the case in gut bacteria in other species with similar implications for longer infectivity and maintenance of the virus source. Given the predominantly gastrointestinal manifestations of an array of other, animals coronaviruses, ^36^ this is an even more pertinent question for SARS-CoV-2.

We are fully aware that a ‘bacteriophage-like’ behaviour of SARS-CoV-2 has not been observed or described before, and that it is in contrast with the current knowledge of virology and molecular biology. It can’t be excluded that behind our observations and results there are new biological mechanisms not yet explored, such as those involving the interaction between anaerobic commensals of human gut and animal viruses through prokaryotic microvesicles, ^37^ evidently poorly studied. For this reason, we believe the findings presented in this work should be subject to controversy and verification by the scientific community. We invite other teams to verify whether they can reproduce results of this nature.

## Conclusions

Severe acute respiratory syndrome coronavirus 2 (SARS-CoV-2), the virus that causes coronavirus disease 2019 (COVID-19), and was responsible for the COVID-19 pandemic is so far considered to be able to infect only eukaryotic cells. Here we report experimental evidence that it can also infect bacteria, specifically two commensal gut bacterial species normally present in healthy human gut microflora. These results generate a wide range of important consequences for the epidemiology, ecology and public health aspect of the virus. We hope our results will contribute for a rethinking of SARS-CoV-2 biology and inform a more effective management and control in case of resurgence of COVID-19 pandemic.

## Data availability

### Underlying data

Zenodo: Underlying data of “Evidence of SARS-CoV-2 bacteriophage potential in human gut microbiota https://doi.org/10.5281/zenodo.5905965. ^38^

The project contains the following underlying data:

• SupplementaryFigureS1.png (Virus-like particles on bacteria surface analysed by STEM-EDX technique.)
• SupplementaryFigureS2.png (Overviews on the quality of the sequencing experiments).
• SupplementaryFile01.fa (File with consensus sequences in fastA format).
• SupplementaryFile02.txt (File with alignment of the obtained consensus sequences to SARS-CoV-2 reference sequence -,NCBI Reference Sequence: NC_045512.2).
• RawFigures.tgz (compressed file with the raw unedited uncropped microscopy images).
• ID2041-1-3992_S90_L001_R1_001.fastq.gz (compressed file with raw R1 reads from *Dorea formicigenerans* plaque).
• ID2041-1-3992_S90_L001_R2_001.fastq.gz (compressed file with raw R2 reads from *Dorea formicigenerans* plaque).
• ID2041-2-17677_S183_L001_R1_001.fastq.gz (compressed file with raw R1 reads from *Faecalibacterium prausnitzii* plaque).
• ID2041-2-17677_S183_L001_R2_001.fastq.gz (compressed file with raw R2 reads from *Faecalibacterium prausnitzii* plaque).

Data are available under the terms of the Creative Commons Attribution 4.0 International license (CC-BY 4.0).

Accession numbers

This project used the following NCBI taxonomy and reference sequences:

• NCBI taxonomy: Faecalibacterium prausnitzii Accession number txid853
• NCBI taxonomy: Dorea formicigenerans Accession number txid39486
• NCBI Protein: surface glycoprotein [Severe acute respiratory syndrome coronavirus 2]. https://identifiers.org/ncbiprotein:YP_009724390.
• NCBI Protein: Severe acute respiratory syndrome coronavirus 2 isolate Wuhan-Hu-1, complete genome. https://identifiers.org/ncbiprotein:NC_045512
