# Supplementary material for: Evidence of SARS-CoV-2 bacteriophage potential in human gut microbiota
Source: F1000Res. 2025 Apr 23;11:292. Originally published 2022 Mar 9. [Version 2] doi: 10.12688/f1000research.109236.2 (PMC12120431; doi:10.12688/f1000research.109236.2)
Supplement: Peer review history

# Reviewer report: Reviewer response for version 2

**DOI:** 10.5256/f1000research.180605.r380905

**Published:** 2025-05-28

**Authors:** Zhonghan Sun

**Affiliations:** 1 Fudan University, Shanghai, Shanghai, China

In this article, Mauro et.al reported that SARS-CoV-2 may behave in a bacteriophage-like manner by infecting and replicating within certain human gut commensal bacteria. The authors present electron microscopy imaging, stable isotope labeling mass spectrometry, and plaque assays to support this hypothesis. While the research question is bold and potentially impactful if substantiated, several aspects of the methodology, data interpretation, and controls need to be more rigorously addressed before strong conclusions can be drawn. 

 Major Comments:

 1. The claim that SARS-CoV-2 replicates in gut bacteria is bold and unconventional. While the authors observed some viral protein signals and genome fragments in bacterial cultures, the current data cannot fully exclude alternative explanations such as uptake of viral RNA, protein adsorption, or contamination.

 2. The evidence for de novo synthesis of spike protein in bacterial cultures is primarily based on ^15N-labeled peptides detected by MS. However, key supporting data such as MS/MS spectra, peptide confidence scores, and comparison with spike standard spectra are not shown.

 3. The presence of virus-like particles on bacterial surfaces and within cells is interesting. Yet, without quantitative analysis or consistent morphology, the conclusions drawn from EM remain speculative.

 4. The study would benefit from additional negative controls to better exclude passive adsorption or contamination.

 5. The conclusion that SARS-CoV-2 replicates in gut bacteria and exhibits bacteriophage-like behavior is not fully supported by the current evidence. A more cautious interpretation is warranted.

Is the work clearly and accurately presented and does it cite the current literature?

Partly

If applicable, is the statistical analysis and its interpretation appropriate?

Not applicable

Are all the source data underlying the results available to ensure full reproducibility?

Yes

Is the study design appropriate and is the work technically sound?

Partly

Are the conclusions drawn adequately supported by the results?

Partly

Are sufficient details of methods and analysis provided to allow replication by others?

Yes

Reviewer Expertise:

Microbiology, cardiometabolic disease

I confirm that I have read this submission and believe that I have an appropriate level of expertise to confirm that it is of an acceptable scientific standard, however I have significant reservations, as outlined above.

# Reviewer report: Reviewer response for version 1

**DOI:** 10.5256/f1000research.120713.r371575

**Published:** 2025-03-28

**Authors:** Didier Raoult

**Affiliations:** 1 Institut Hospitalo-Universitaire Méditerranée-Infection, Faculté de médecine, Aix-Marseille Université, URMITE, Marseille, France

This work is very interesting, but it overturns a well-established theory about the host specificity of viruses. The idea that a mammalian eukaryotic cell virus could multiply in bacteria in a way comparable to bacteriophages is a staggering one. The techniques employed by the authors are comprehensive, ranging from electron microscopy to nucleotide sequence, proteomic evaluation and in situ hybridization. Technically, this work is indisputable. The only thing that could call into question the quality and significance of this work would be the existence of a fraud that I am unable to detect. In practical terms, I think it's very important to accept this work and subject it to controversy and verification by other scientific teams. With this in mind, our team will be moving quickly to verify whether we can reproduce results of this nature.

Is the work clearly and accurately presented and does it cite the current literature?

Yes

If applicable, is the statistical analysis and its interpretation appropriate?

Not applicable

Are all the source data underlying the results available to ensure full reproducibility?

Yes

Is the study design appropriate and is the work technically sound?

Yes

Are the conclusions drawn adequately supported by the results?

Yes

Are sufficient details of methods and analysis provided to allow replication by others?

Yes

Reviewer Expertise:

Microbiology

I confirm that I have read this submission and believe that I have an appropriate level of expertise to confirm that it is of an acceptable scientific standard.

# Response

**Published:** 2025-04-12

**Authors:** Mauro Petrillo

**Affiliations:** European Commission, Joint Research Centre (JRC), Ispra, Italy

Dear Dr. Raoult, 

 ​​​​​​We thank you for your open-mindedness and for encouraging a broader dialogue around this work. We are committed to scientific transparency, and we are pleased to read that the reviewer’s team intends to attempt replication of our results. We believe that only through open scientific discourse and independent validation can the implications of such findings be fully understood and either confirmed or refuted. For this reason, the following sentence has been added at the end of the paper: “ *We are fully aware that a ‘bacteriophage-like’ behaviour of SARS-CoV-2 has not been observed nor described before, and that it is in contrast with the current knowledge of virology and molecular biology. It can’t be excluded that behind our observations and results there are new biological mechanisms not yet explored, such as those involving the interaction between anaerobic commensals of human gut and animal viruses through prokaryotic microvesicles [https://doi.org/10.1186/s12964-023-01414-8], evidently poorly studied. For this reason, we believe the findings presented in this work should be subject to controversy and verification by the scientific community. We invite other teams to verify whether they can reproduce results of this nature.*”

 Thank you again for your thoughtful evaluation and the opportunity to improve our work.

 Sincerely,

 Mauro Petrillo,

 On behalf of all authors

# Reviewer report: Reviewer response for version 1

**DOI:** 10.5256/f1000research.120713.r154688

**Published:** 2022-11-25

**Authors:** M. Anwar Hossain, Md. Shaminur Rahman

**Affiliations:** 1 Department of Microbiology, University of Dhaka, Dhaka, Bangladesh; 2 Department of Microbiology, Jashore University of Science and Technology, Sadhinata Sarak, Bangladesh

This work is nicely designed but only two bacterial strains ( *Faecalibacterium prausnitzii* and *Dorea formicigenerans* strain) were tested here.

 The Major comments are listed below:

1. On which basis these two bacteria were taken? 
2. " *Faecalibacterium prausnitzii* and *Dorea formicigenerans* strains were purchased at the Leibniz Institute DSMZ-German Collection of Microorganisms and Cell Cultures"; Did they confirm that these bacteria were also present in the human gut from where they collected samples?
3. Through NGS, they sequenced the SARS-CoV-2 genome. **"** ***host specific” nucleic acid changes"*** Did the author sequence these genomes before inoculation to the bacterial culture? This variation might be the different variants of SARS-CoV-2 variants as these were collected from two different suspected patients. What evidence the author is going to give here as the genome changing inside the multiplication of bacterial strains?
4. Bacteriophage potential is showing only the two isolates? What will happen if E. coli is used here?
5. Add discussion on which mechanisms the virus multiplies inside prokaryotic cells as we know it is an animal virus.
6. Clear figure legends from Figure 1 to figure 4. The author can include here the name of the isolates.
7. Submit those genome and raw sequence file to NCBI or other repository.

Is the work clearly and accurately presented and does it cite the current literature?

Yes

If applicable, is the statistical analysis and its interpretation appropriate?

Not applicable

Are all the source data underlying the results available to ensure full reproducibility?

Yes

Is the study design appropriate and is the work technically sound?

Partly

Are the conclusions drawn adequately supported by the results?

Yes

Are sufficient details of methods and analysis provided to allow replication by others?

Yes

Reviewer Expertise:

Virology, Genomics, Metagenomics, Vaccine Development

We confirm that we have read this submission and believe that we have an appropriate level of expertise to confirm that it is of an acceptable scientific standard, however we have significant reservations, as outlined above.

# Response

**Published:** 2022-11-25

**Authors:** Mauro Petrillo

**Affiliations:** European Commission, Joint Research Centre (JRC), Ispra, Italy

Dear M. Anwar Hossain and Md. Shaminur Rahma,

 Thanks a lot for your valuable comments and suggestions that you have provided in the report.

 We will address all of them, and wait for those of other reviewers, in order to provide a fully revised version of the manuscript.

 Best regards,

 Mauro Petrillo, on behalf of the authors.

# Response

**Published:** 2025-04-12

**Authors:** Mauro Petrillo

**Affiliations:** European Commission, Joint Research Centre (JRC), Ispra, Italy

Dear Dr. Anwar Hossain and Dr. Md. Shaminur Rahman,

 We sincerely thank you for the constructive feedback on our manuscript. We appreciate the opportunity to clarify and revise our work. Below, we provide detailed responses to each of the comments raised.

**Comment 1: **“On which basis these two bacteria were taken?”

*Response:* *As we indicated in the text, the selection of Faecalibacterium prausnitzii and Dorea formicigenerans was justified by the fact that both had previously been reported as being strongly reduced in COVID-19 patients (Ref. 17 and Ref. 18). We rephrased the Results section to better clarify this point.*

**Comment 2:** “Did they confirm that these bacteria were also present in the human gut from where they collected samples?”

*Response:* *Thank you for this comment. We did not check whether these two species were present in our collected samples. However, these bacteria are common gram-positive anaerobic commensals in the human gut, and thus it is likely they were also present in the faecal samples collected and used in this study. We have clarified this in the Discussion section by revising the text as follows: “It is important to highlight that this study is limited to SARS-CoV-2 interaction with only two bacterial strains, i.e., F. prausnitzii and D. formicigenerans, chosen because both were previously reported as strongly reduced in COVID-19 patients ^17,18^. These are common gram-positive anaerobic commensals in the human gut, and thus it is likely they were also present in the faecal samples collected and used in this study.”*

**Comment 3:** “Through NGS, they sequenced the SARS-CoV-2 genome. "host specific” nucleic acid changes" Did the author sequence these genomes before inoculation to the bacterial culture? This variation might be the different variants of SARS-CoV-2 variants as these were collected from two different suspected patients. What evidence the author is going to give here as the genome changing inside the multiplication of bacterial strains?”

*Response:* *Thank you for the correct observation.  We revised the text and replaced the term “host specific” with “sample-specific”. In addition, we revised the Discussion section in which this point is discussed by adding a sentence, here underlined as follows: “In our experiments, these observations might be either a consequence of different lineages already present in the supernatant aliquots of infected bacteria used in the plaque assay, or a result of the two different bacterial “cellular machinery” environments, which the virus used when infecting two different species. Both consequences cannot be excluded. However, the presence of many identified variations not specific of any lineage provides an indication that bacteria might be a potential source of novel SARS-CoV-2 mutations, and gives rise to the possibility that intra-host SARS-CoV-2 haplotypes might reflect different intestinal bacterial prior host environments of the virus.”*

**Comment 4: **“Bacteriophage potential is showing only the two isolates? What will happen if E. coli is used here?”

*Response:* *We appreciate this suggestion. Our study was a preliminary investigation focusing on human gut-resident commensals and E. coli was not included in the study. However, we acknowledge the value of using E. coli as a model organism in future studies to generalise the findings and explore potential broader bacteriophage-like interactions. This has been discussed in the revised Discussion section, by adding the following sentence: “It is important to highlight that this study is limited to SARS-CoV-2 interaction with only two bacterial strains, i.e., F. prausnitzii and D. formicigenerans, chosen because both were previously reported as strongly reduced in COVID-19 patients ^17,18^. These are common gram-positive anaerobic commensals in the human gut, and thus it is likely they were also present in the faecal samples collected and used in this study. The interaction of SARS-CoV-2 with other common commensals, such as the facultative anaerobic gram-negative Escherichia coli should be investigated, too.”*

**Comment 5:** “Add discussion on which mechanisms the virus multiplies inside prokaryotic cells as we know it is an animal virus.”

*Response:* *This is a critical and insightful comment. While SARS-CoV-2 is known and generally considered an animal virus, our results suggest a potential unconventional interaction or RNA persistence within bacterial environments. We think that speculative considerations on this go beyond the scope of the paper. However, we added this final sentence at the end of the Discussion section, to explicitly state that further mechanistic work would be needed: “We are fully aware that a ‘bacteriophage-like’ behaviour of SARS-CoV-2 has not been observed nor described before, and that it is in contrast with the current knowledge of virology and molecular biology. It can’t be excluded that behind our observations and results there are new biological mechanisms not yet explored, such as those involving the interaction between anaerobic commensals of human gut and animal viruses through prokaryotic microvesicles [https://doi.org/10.1186/s12964-023-01414-8], evidently poorly studied. For this reason, we believe the findings presented in this work should be subject to controversy and verification by the scientific community. We invite other teams to verify whether they can reproduce results of this nature.”*

**Comment 6:** “Clear figure legends from Figure 1 to figure 4. The author can include here the name of the isolates.”

*Response:* *Thank you for the suggestion. We have revised all figure legends and added clarifying details to improve readability and interpretation.*

**Comment 7:** “Submit those genome and raw sequence file to NCBI or other repository.”

*Response:* *Thank you for the suggestion. We have now integrated raw sequencing data as part of the Underlying data in Zenodo: Underlying data of “Evidence of SARS-CoV-2 bacteriophage potential in human gut microbiota”, available at https://doi.org/10.5281/zenodo.5905965.*

 We hope these revisions address your concerns and enhance the clarity and impact of our manuscript. Thank you again for your thoughtful evaluation and the opportunity to improve our work.
